# The epidemiological landscape of canine tumors in central Italy: animal cancer registry insights, distribution patterns, and incidence estimates

**DOI:** 10.3389/fvets.2026.1919722

**Published:** 2026-09-08

**Authors:** Azzurra Carnio, Cristiano Cocumelli, Valentina Galietta, Niccolò Fonti, Sara Simeoni, Marta Barberini, Valentina Panetta, Paola Scaramozzino, Francesca Millanta, Claudia Eleni, Andrea Carvelli

**Affiliations:** 1 Istituto Zooprofilattico Sperimentale del Lazio e della Toscana “M. Aleandri”, Rome, Italy; 2 Department of Comparative Biomedicine and Food Science, University of Padova, Legnaro, PD, Italy; 3 L'altrastatistica srl - Consultancy and Training- Biostatistics Office, Rome, Italy; 4 Department of Veterinary Sciences, University of Pisa, Pisa, Italy

**Keywords:** cancer registry, dog, incidence, population estimation, Proportional Morbidity Ratio, retrospective analysis, tumor

## Abstract

**Background:**

Animal cancer registries (ACRs) monitor companion animal tumors, providing essential data for comparative oncology. Sharing domestic environments and possessing compressed lifespans, dogs serve as “One Health” environmental sentinels.

**Objectives:**

This study aimed to provide a descriptive analysis of canine neoplasms recorded in the ACR of the Lazio Region (central Italy) over 15-year period (2009–2023) and generate a survey-adjusted crude incidence estimate.

**Methods:**

Primary tumors were standardized using Vet-ICD-O-canine-1. Proportional Morbidity Ratios (PMRs) were used to evaluate associations between demographic and environmental characteristics and the relative distribution of malignant tumor topographies. Incidence estimates were calculated using an adjusted canine population and corrected tumor counts to account for under-ascertainment. The population denominator was adjusted using a validated 75.3% registry completeness factor to calculate the annual crude incidence estimate.

**Results:**

A total of 9,823 primary tumors from 9,122 dogs were included. Malignant tumors accounted for 59.3% of cases. Skin (38.4%), mammary gland (23.2%), and soft tissues (13.3%) were the most frequent topographies. The highest proportion of cases was observed in dogs aged 8–11 years. Among cases, 60.2% were purebred, 58.3% were fed industrial diets, and 49.1% lived primarily indoors. Females accounted for 57.7% of cases, although sex-stratified age-adjusted PMR analysis showed higher proportions of most tumors in males, except for mammary tumors (adj PMR = 46.22; 95% CI: 29.75–71.81). Intact females showed higher PMRs for mammary and female genital organs tumors, whereas spayed females had higher PMRs for hematopoietic, skin, digestive, and nervous system tumors. Higher PMRs for skin tumors were observed in dogs fed industrial diets, while environmental factors showed associations with specific tumor sites, including a higher frequency of skin tumors in outdoor dogs and a higher proportional frequency of mammary tumors in indoor dogs. The estimated crude incidence was 662.1 cases per 100,000 dogs, including 249.4 malignant and 412.7 benign tumors per 100,000 dogs.

**Conclusions:**

The ACR represents a valuable tool for monitoring canine cancer and exploring potential determinants of tumor occurrence. The integration of registry and survey data provided adjusted incidence estimates and improved the assessment of tumor burden. The findings support the use of ACRs in epidemiological surveillance and One Health research.

## 1 Introduction

Animal cancer registries (ACRs) represent a key tool for the systematic collection, storage and management of data on animal tumors. Much like human cancer registries, an ACR enables the identification of potential environmental, biological, and anthropogenic determinants of carcinogenesis. The primary objective of a cancer registry (CR) is the registration of all cancer cases occurring in a defined population, collecting information on demographic characteristics of patients and on tumor features. From a public health perspective, periodic analyses of registry data provide information on cancer incidence, temporal variations, and geographic clustering, contributing to the identification of potential risk factors (1, 2). Besides their role in toxicology, animals act as invaluable sentinels for chronic and neoplastic diseases, as they have shorter lifespans and latency periods than humans while being exposed to the same xenobiotics and potential carcinogens (2, 3). Although genetic predisposition remains a key determinant of cancer risk across species, environmental and lifestyle factors (e.g., diet, habitat, living environment) interact dynamically within a multistage evolutionary model of carcinogenesis (4). Dogs develop tumors spontaneously, are relatively outbred, immunocompetent, and tumor initiation and progression are influenced by age, diet, sex, and reproductive status in canine species. Consequently, shifts in tumor incidence or morphological patterns can be detected significantly earlier in canine populations than in human cohorts. The expanding field of comparative oncology leverages these dynamics to benefit both veterinary and human medicine (2, 3, 5–7).

In Italy, the first ACR was set up in Genoa in 1985 (8). Currently, there are several independent ACRs nationwide, that implement the Italian Network of Laboratories for Veterinary Oncology (NILOV), a national database on canine and feline tumors, coordinated by the National Reference Centre (9). The ACR of the Lazio Region was established in 2009 at the Istituto Zooprofilattico Sperimentale del Lazio e della Toscana “M. Aleandri” (IZSLT). It collects data on dog and cat neoplasms diagnosed by the IZSLT laboratory with additional voluntary contributions from external veterinary facilities or laboratories that were intermittent over time and became systematically structured from 2020 onwards through a strategic research project (PS IZS PLV 2019) funded by the Italian Ministry of Health, involving the University of Pisa (UNIPI), private laboratories, and veterinary practitioners actively submitting tumor data on oncological cases. As a laboratory-based surveillance system, the ACR gathers data differently from a population-based cancer registry (PBCR), introducing inherent challenges such as the under-reporting of cases (10) and potential ascertainment biases. For instance, the decision to seek veterinary care can be influenced by the socioeconomic status of the owner and proximity to a veterinary facility (11–13). As a result, these factors contribute to under-ascertainment, in that the diagnostic examination is not performed at all (10) or, if cancer is diagnosed, it may not be captured within the operational coverage of the ACR. Furthermore, the lack of a comprehensive census of animals (denominator) is a major limitation for developing population-based veterinary cancer registries (1, 9, 14, 15). Italy, however, presents a unique epidemiological advantage: a mandatory National Canine Population Database has been in place since 1991, and its completeness within the Lazio Region has been rigorously validated (16, 17). This population baseline allows for robust incidence testing despite underlying registry biases. Oncological data reporting in companion animals is not mandatory under current legislation, but different targeted initiatives have been implemented over the years by the ACR of Lazio. These included providing free anatomo-histopathological diagnostics for suspected neoplasms, active data collection directly at private veterinary facilities, and collection of data from private practitioners to provide data on oncological cases diagnosed by other laboratories. These tasks have been promoted throughout the Lazio Region, thanks to research projects founded by the Italian Ministry of Health (IZSLT RC 04/17; IZSPLV PS 2019; IZSLT RC 05/19). These efforts aim to provide a reliable estimate of canine tumor incidence at the regional level. As reported by Fonti and Millanta (18), working groups from different countries have attempted to estimate the incidence of neoplasms in pets. Incidence estimates retrieved in the scientific literature from 1968 to 2024 ranged from 16.0 to 4,817 per 100,000 dog-years at risk, with a wide variability in diverse methodological approaches used, that ultimately affects the comparability between estimates.

The purpose of the present study was to provide a comprehensive descriptive analysis of canine neoplasms recorded in the ACR of Lazio over a fifteen-year period (2009–2023), evaluating case distribution by sex, age, and tumor site, and to generate a survey-adjusted regional crude incidence estimate for overall, malignant, and benign tumors. In addition, the study aimed to evaluate the contribution of integrating registry and survey-derived data for estimating tumor burden and addressing under-ascertainment within a laboratory-based cancer registry. Finally, these findings are contextualized through comparisons with other animal cancer registries and with human and canine population data from the same geographical territory, highlighting the potential value of integrated animal and human health data for comparative epidemiological investigations within a One Health framework.

## 2 Materials and methods

### 2.1 The study area

The study area encompasses the Lazio Region, which is divided into five provinces: Frosinone, Latina, Rieti, Rome, Viterbo (Table 1), spanning a total of 378 municipalities (45). Human population data were obtained from Istituto Nazionale di Statistica (ISTAT, accessed in 2026) (19). Between 2011 and 2023, the regional human population ranged from 5.5 to 5.7 million inhabitants, with marked demographic concentration in the Province of Rome, which accounts for approximately 74.0% of the regional population, reflecting its high level of urbanization compared to the predominantly rural areas in the other provinces. Canine population data were retrieved from the Regional Canine Registry (Anagrafe Canina Regionale del Lazio). In the reference year 2021, a total of 807,556 dogs were registered in the region. To contextualize the geographic distribution of cases, the absolute frequencies and proportions of human residents, registered dogs, and recorded tumor cases were evaluated across the five provinces (Table 1), allowing evaluation of potential discrepancies between human and canine population distribution and case reporting.

**Table 1:** Area, absolute frequency and proportion of human residents, registered dogs, and tumor cases in Lazio Region.

Province	Area (km^2^)	Human population		Dog population		Tumors	
		No	%	No	%	No	%
Rome	5,367	4,216,874	74%	520,150	64%	7,290	78%
Latina	2,259	567,439	10%	89,774	11%	1,203	13%
Frosinone	3,247	470,689	8%	83,733	10%	243	3%
Rieti	2,750	151,143	3%	39,335	5%	122	1%
Viterbo	3,616	308,737	5%	74,564	9%	458	5%
Total	17,239	5,714,882	100%	807,556	100%	9,316	100%

### 2.2 Data source and standardization

This retrospective study analyzed benign and malignant primary tumors diagnosed in dogs residing in the Lazio Region (central Italy) and recorded in the regional Animal Cancer Registry (ACR) between 2009 and 2023. For each tumor, a standardized set of metadata was recorded, including: tumor diagnosis (morphology) and localization (topography), animal id (microchip number or name), address and municipality, date of birth or age at diagnosis, breed, sex, neuter status, body size, weight, main diet, general condition, living environment and habitat.

Data collection relied on a dedicated submission form completed by veterinary practitioners when sending tissue specimens or animal carcasses to the Istituto Zooprofilattico Sperimentale del Lazio e della Toscana (IZSLT) or the University of Pisa (UNIPI) laboratories for histopathological or necropsy examination. The same form was utilized to report oncological cases diagnosed by external private laboratories. Histopathological diagnoses performed at the IZSLT and UNIPI facilities were established according to the International Histological Classification of Tumors of Domestic Animals (20, 21). Diagnoses referred by external private practitioners were accepted as originally reported.

To ensure independent tumor events for the descriptive analysis, multiple tumors presenting the same histotype and topography in the same animal were considered a single event, whereas multiple synchronous or metachronous neoplasms of different histotypes or anatomical sites were recorded separately. Metastatic and recurrent lesions were excluded from the analysis dataset. Topographic and morphological features were coded using the Vet-ICD-O-canine-1 classification system (22). Tumors were subsequently grouped into 20 topographic categories following the criteria proposed by Dhein et al. (23) and Fonti et al. (24). The data cleaning process followed the procedure described by Fonti et al. (24), ensuring consistency and accuracy of the dataset prior to analysis. According to the Vet-ICD-O-canine-1 coding system, tumors were classified as malignant, benign, or of uncertain behavior, following the standardized malignancy criteria proposed by Pinello et al. (22) and adopted by Fonti et al. (24). Age at diagnosis was calculated by subtracting the date of birth from the date of sampling and grouped into five age classes (0–3 years, 4–7 years, 8–11 years, 12–15 years, >15 years). Dogs were classified into purebred and non-purebred.

### 2.3 Proportional Morbidity Ratio (PMR)

Metadata were initially explored through descriptive summaries, and temporal trend was assessed based on annual counts of recorded tumors. To investigate whether the frequency of specific malignant tumor sites (topography) varied according to selected characteristics, we estimated the Proportional Morbidity Ratio (PMR), as previously described (9, 25, 26). The selected variables included: sex, reproductive status, breed, diet, habitat, living environment, and time period. Analyses used predefined reference categories: male (sex), neutered dogs (reproductive status), non-purebred (breed), homemade diet (diet), rural habitat (habitat), and indoor (living conditions) and 2009–2017 (time period). For each animal, a single diagnosis per tumor site was retained to avoid duplication. PMRs were estimated using two complementary approaches:

(i) Descriptive approach, by computing the PMR as the ratio between the proportion of tumors in the analyzed category and the proportion in the reference category, with 95% confidence intervals (95% CI) assuming Poisson-distributed case counts for the log-scale standard error;
(ii) Model-based approach: Poisson regression models were used to obtain adjusted PMR estimates, providing more robust quantification of the associations; analyses were repeated using Poisson regression models adjusted for age, categorized into five classes ( ≤3, 4–7, 8–11, 12–15, and >15 years), to account for potential confounding.

### 2.4 Tumor incidence

The crude annual incidence of overall, malignant, and benign tumors per 100,000 dogs was estimated using the number of tumor cases as the numerator and the regional canine population as the denominator, with 2021 selected as the cross-sectional reference year. To correct the numerator for underreporting by private veterinarians, a questionnaire-based survey was administered to veterinary facilities across the Lazio Region in 2021. The list of active facilities (*N* = 744) was provided by the Veterinary Local Health Units (LHU), and contact information was supplemented via professional registries and online directories. The survey, structured into 12 open questions across two sections (Table 2), was distributed via Google Forms to collect data regarding facility features (Section A) and average monthly canine caseloads alongside diagnosed oncological cases (Section B).

**Table 2:** Survey questionnaire administered to veterinary facilities of Lazio Region, including information on facility characteristics and canine oncological caseloads.

Section A— Facility data	Section B—Animal and oncological cases data
1. Facility type	4. The mean number of attended dogs in a month
2. Facility denomination	5. The mean number of diagnosed tumors (new cases) in a month (both malignant and benign)
3. City, postcode	6. The mean number of malignant tumors (new cases) diagnosed in a month
	7. The mean number of benign tumors (new cases) diagnosed in a month
	8. Use of the IZSLT histopathological service (yes/no)
	9. Percentage of histopathological samples sent to the IZSLT for the diagnosis
	10. Performance of necropsy (yes/no)
	11. The mean number of necropsies requested in a year
	12. Percentage of necropsy sent to the IZSLT

Based on the survey, we conducted an analysis of tumor submission to IZSLT. The average monthly canine patient volume intended as Visits per month (Visits) and the percentage of total tumors (samples) submitted to IZSLT (%Submission) were considered. The overall submission fraction was calculated using a volume-weighted average to account for the disproportionate caseload across facilities. The formula applied was:


$$\begin{array}{l} Weighted\;Submission\;Rate=\sum \;\frac{\left(Visits_{i\;}\times \;{\%Submission}_{i}\right)}{\sum \left(Visits_{i}\right)} \end{array}$$

This method ensures that the contribution of each structure to the final ratio is proportional to its clinical activity, providing a more accurate picture of the total surveillance coverage.

The denominator was retrieved by extracting the dog population from the regional dog database (reference year: 2021). In Italy, dog registration and identification have been mandatory since 1991 (Law No. 281/1991), and CRs were established and managed at the regional level at the time of the present study. In the Lazio Region, a previous study estimated that 75.3% of dogs were effectively registered (17). This proportion was then used to estimate the corrected regional dog population size, adjusting the denominator to account for unregistered dogs.

To estimate the incidence of malignant and benign tumors, we applied the proportion of each tumor type reported in the survey to the overall incidence. For each tumor type, the reporting proportion was calculated by dividing the number of recorded tumors within the ACR of Lazio by the associated estimated total number.

#### 2.4.1 Sensitivity analysis

To evaluate the robustness of incidence estimates to assumptions derived from the veterinary survey, a sensitivity analysis was performed by varying the estimated sample submission rate. For malignant and benign tumors, the proportions of malignant and benign cases reported in the survey were also varied using the minimum and maximum values observed. The dog population denominator was kept constant using the regional registry completeness factor (75.3%) estimated by Carvelli et al. (17). This parameter was not included in the sensitivity analysis because it was obtained independently from the survey data and was consistent with previous estimates of dog registry completeness reported for the same geographical area by Caminiti et al. (16).

### 2.5 Data analysis

Data management and statistical analyses were performed using Microsoft Excel version 16.84 and Stata Statistical Software: Release 16 (College Station, Texas, StataCorp. LLC.). Geospatial analyses were conducted using ArcGIS Pro version 3.6.2 (Esri, Redlands, CA, USA).

## 3 Results

### 3.1 Descriptive statistics

#### 3.1.1 Oncological cases

**Figure 1:**
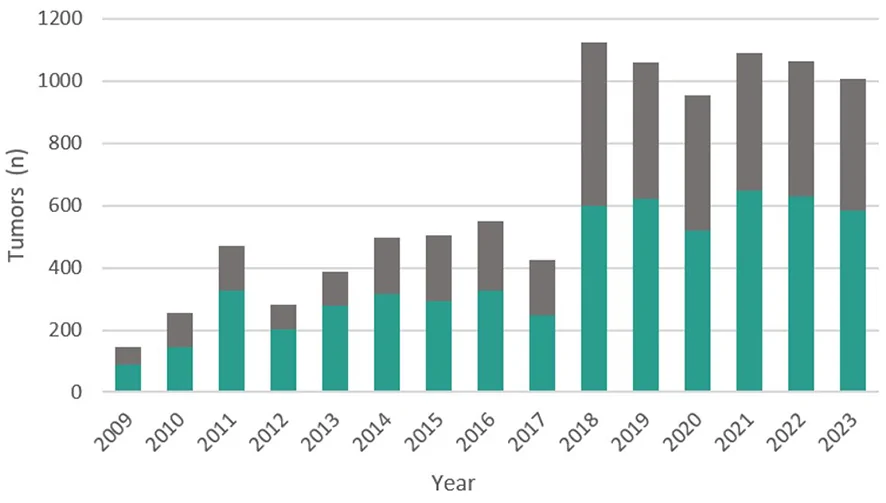
Annual distribution of tumors collected in the ACR of Lazio Region, with stacked bars highlighting the contribution of benign (green) and malignant (gray) tumor cases.

Between 2009 and 2023, the ACR of Lazio recorded a total of 11,527 oncological diagnoses, including metastases and recurrences, corresponding to 10,324 dogs. After coding and data cleaning, metastatic and recurrent lesions were excluded, and 9,823 tumors in 9,122 dogs were retained for analysis. Malignant tumors accounted for 59.3% (5,821/9,823) of all neoplasms. Most diagnoses originated from biopsy samples (96.9%; 9,523/9,823), while the remaining were obtained from necropsies. The analysis of the temporal trend highlights a marked increase in the number of registered tumors starting from 2018. In the period 2009–2017 the number of annual cases ranged between 147 and 551 tumors, while since 2018 over 1,000 cases have been recorded in almost all years (Figure 1).

#### 3.1.2 Geographical pattern

**Figure 2:**
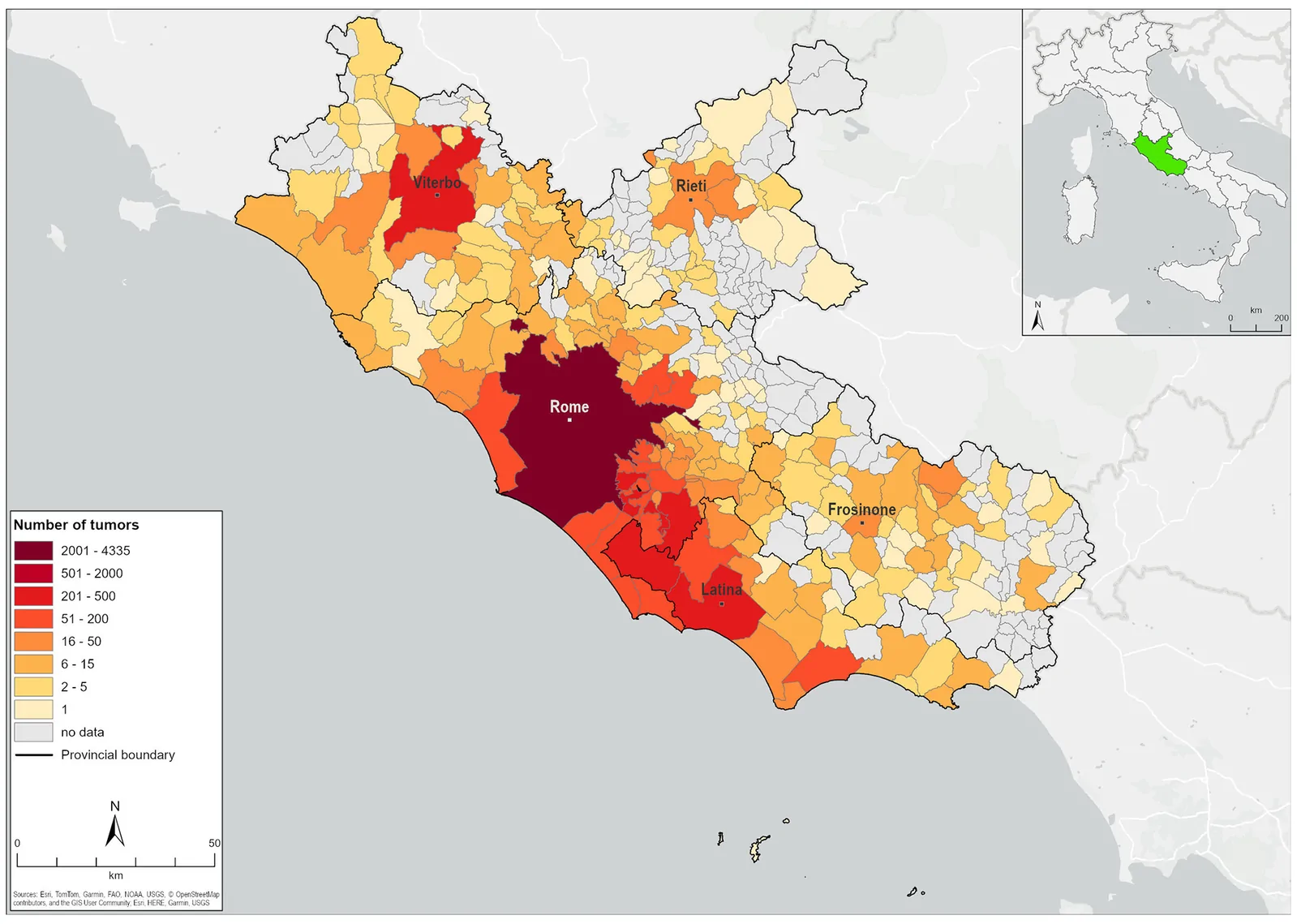
Choropleth map illustrating the spatial distribution of canine tumor cases recorded in the Animal Cancer Registry (ACR) of the Lazio Region across municipalities.

The spatial distribution across 378 municipalities of 9,316 tumors (507 missing data) using a choropleth map (Figure 2) reflects a highly skewed pattern of cases across the Lazio Region; for this reason, broader classes were applied to the upper end of the distribution and narrower classes to the lower end. Tumors were identified in 234 municipalities (61.9% of the regional total), strongly mirroring human and canine population densities. Urban centers and provincial capitals demonstrated prominent concentrations of cases, with the metropolitan area of Rome exhibiting the largest cluster. Analysis by administrative province confirmed that Rome contributed the highest volume of cases (7,290), followed by Latina (1,203), Viterbo (458), Frosinone (243), and Rieti (122) (Table 1).

#### 3.1.3 Demographic characteristics

Demographic profiling of the affected population (Tables 3, 4) showed a higher representation of females (57.7%; 5,261/9,122). Intact animals accounted for 61.3% (5,589/9,122) of the total, but differences were found when considering females and males separately. Among females, the distribution by neuter status was almost equal (49.1% intact females; 49.8% neutered females; 1.1% not reported). Differently, intact male dogs were more frequent than the neutered ones (79.2% intact males; 19.7% neutered males; 1.0% not reported). Age at the diagnosis was available for 95.6% (8,723/9,122) of the dogs, showing a mean age at diagnosis of 8.8 years (SD = 3.4). Considering age categories, the most frequent age group was 8–11 years (43.9%; 4,000/9,122), followed by the 4–7 years (24.0%; 2,191/9,122) and 12–15 years age groups (19.4%; 1,769/9,122). Regarding breed distribution, purebred dogs were the most represented (60.2%; 5,496/9,122). Most dogs were fed industrial diets (58.3%; 5,318/9,122), followed by mixed diets combining commercial and homemade foods (11.4%; 1,044/9,122); nutritional information was unavailable for 22.7% of dogs. Dogs lived primarily indoors (49.1%; 4,482/9,122), whereas 22.6% (2,062/9,122) of dogs were reported to live predominantly outdoors; only 12.6% (1,154/9,122) had access to both indoor and outdoor environments. Most tumor cases were reported in dogs residing in urban areas (61.8%; 5,633/9,122), followed by rural (16.9%; 1,550/9,122) and semi-urban environments (3.4%; 311/9,122).

**Table 3:** Demographic characteristics of dogs with neoplasms in the ACR of Lazio Region, 2009–2023.

Variables		Animals (*N* = 9,122)
Sex, *n* (%)	Female	5,261 (57.7%)
	Male	3,792 (41.6%)
	Not reported	69 (0.7%)
Age in year, mean (sd)		8.82 (3.4)
Age in classes, *n* (%)	≤ 3	637 (6.9%)
	[4–7]	2,191 (24.0%)
	[8–11]	4,000 (43.9%)
	[12–15]	1,769 (19.4%)
	>15	126 (1.4%)
	Not reported	399 (4.4%)
Breed, *n* (%)	Purebred	5,496 (60.2%)
	Non-purebred	3,426 (37.6%)
	Not reported	200 (2.2%)
Period, *n* (%)	2009–2017	3,331 (36.5%)
	2018–2023	5,791 (63.5%)

**Table 4:** Sex and reproductive status among dogs with neoplasms in the ACR of Lazio Region, 2009–2023.

Variables		Female (*N* = 5,261)	Male (*N* = 3,792)	Overall^*^(*N* = 9,122)
Reproductive status, *n* (%)	Intact	2,584 (49.1%)	3,005 (79.3%)	5,589 (61.3%)
	Neutered	2,619 (49.8%)	748 (19.7%)	3,367 (36.9%)
	Not reported	58 (1.1%)	39 (1.0%)	166 (1.8%)

^*^The “Overall” column includes all dogs enrolled in the study (*N* = 9,122), including individuals for which sex was not reported.

A subgroup of 621 animals had multiple tumors at the time of diagnosis, ranging from two to five neoplasms. Of these, 43.6% presented both benign and malignant tumors concurrently, while 30.0% and 26.4% of animals had only malignant or only benign tumors, respectively. Preliminary evidence from our dataset also identified cases with multiple malignant primary tumors, which were further investigated separately using specific epidemiological criteria (27).

#### 3.1.4 Neoplasms frequencies

**Figure 3:**
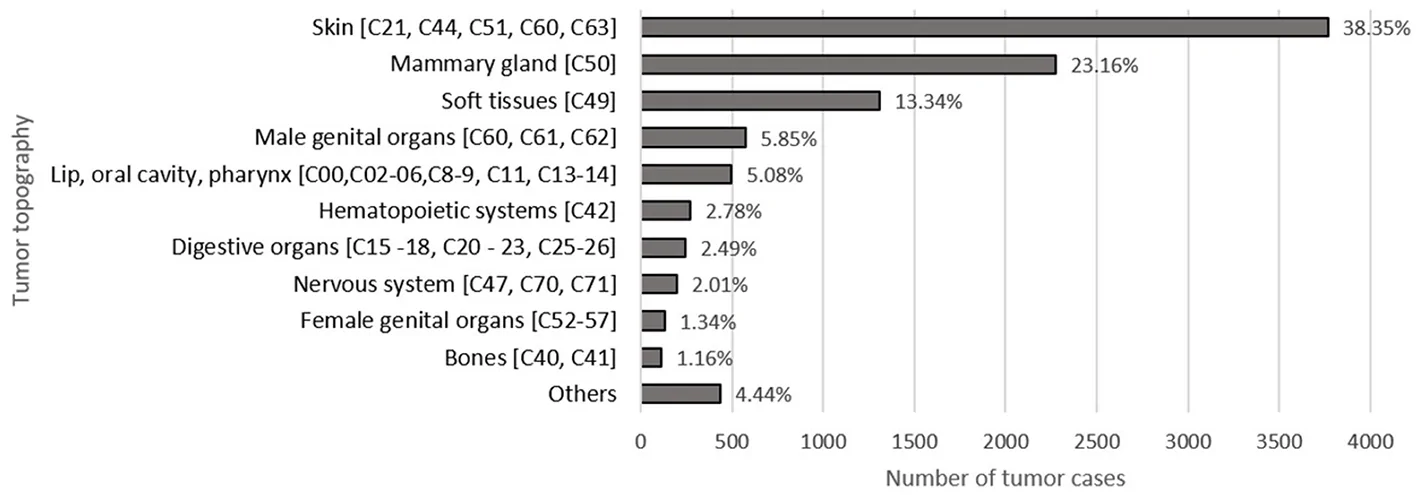
The most frequent tumor topographies (≥1.0%), among all tumors registered in the ACR of Lazio, 2009–2023. C60 was included in different topography categories depending on the diagnosis (morphology).

The most frequent topography sites were the skin [C21, C44, C51, C60, C63], accounting for 38.4% (3,767/9,823) of all cases, followed by the mammary gland [C50] (23.2%; 2,275/9,823), and soft tissues [C49] (13.3%; 1,310/9,823). Figure 3 summarizes the distribution of the most common topographic sites recorded in the ACR.

**Figure 4:**
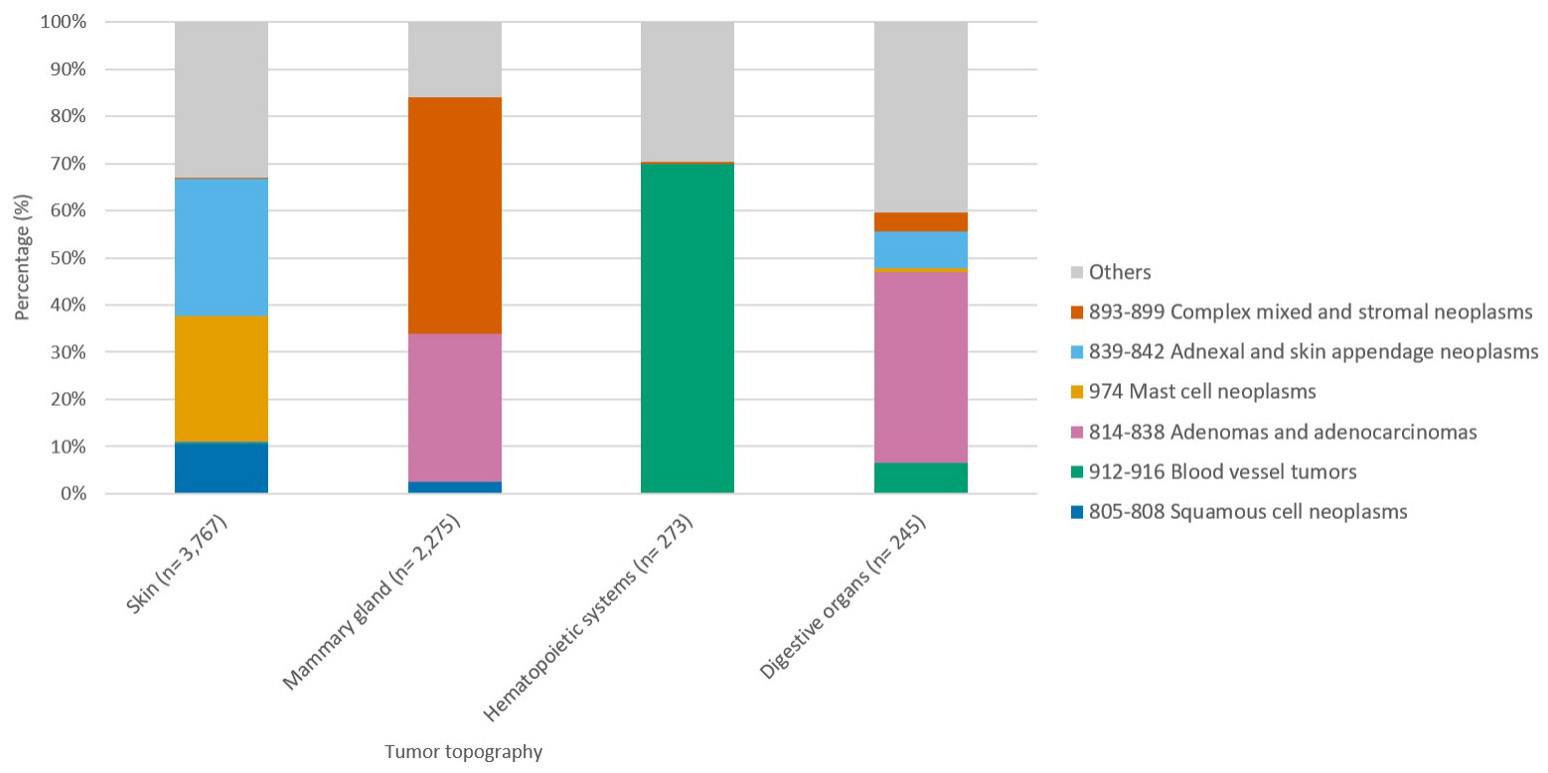
Percentage distribution of the six most frequent tumor morphologies across selected topography site in the ACR of Lazio. Stacked bars represent the relative contribution of each morphology. Only topographic sites in which the residual category “Others” accounted for ≤40% of tumor cases are shown. Additional anatomical topographic sites and less frequent tumor diagnoses (≥1%) are reported in Supplementary Table S1.

The most frequent morphology groups within the skin topography [C21, C44, C51, C60, C63] were adnexal and skin appendage neoplasms [839–842] (29.09%; 1,096/3,767) and mast cell neoplasms [974] (26.6%; 1,001/3,767), which together accounted for 55.7% of all diagnoses in this category; while for soft tissues topography [C49], the most common morphology groups were lipomatous neoplasms [885–888] (28.1%; 368/1,310) and blood vessel tumors [912–916] (27.48%; 360/1,310). Figure 4 shows the percentage distribution of the six most frequent tumor morphologies across selected anatomical sites recorded in the ACR of Lazio. To ensure interpretability of the graphical representation, only topographies in which the residual category “Others” accounted for no more than 40% of tumor cases were included in the figure. Based on this criterion, four anatomical sites (skin, mammary gland, hematopoietic system, and digestive organs) were selected. Across all included sites, the same major tumor morphologies were observed, although their relative contributions varied markedly by anatomical site, resulting in different proportions of residual diagnoses. Tumor diagnoses across additional anatomical topographic sites with an overall frequency ≥1.0%, as well as less frequent morphologies, are reported in Supplementary Table S1.

### 3.2 Proportional Morbidity Ratio (PMR)

The crude and age-adjusted PMR analyses on malignant tumors produced similar results. In this section we reported only the age-adjusted PMR. The analyses were restricted to the 10 most frequently observed topographies. Since the study is ACR-based and lacks information on healthy dogs within the Lazio Region with comparable demographic and exposure characteristics, it is not possible to identify risk or protective factors from the variables analyzed; instead, only associations can be highlighted.

**Figure 5:**
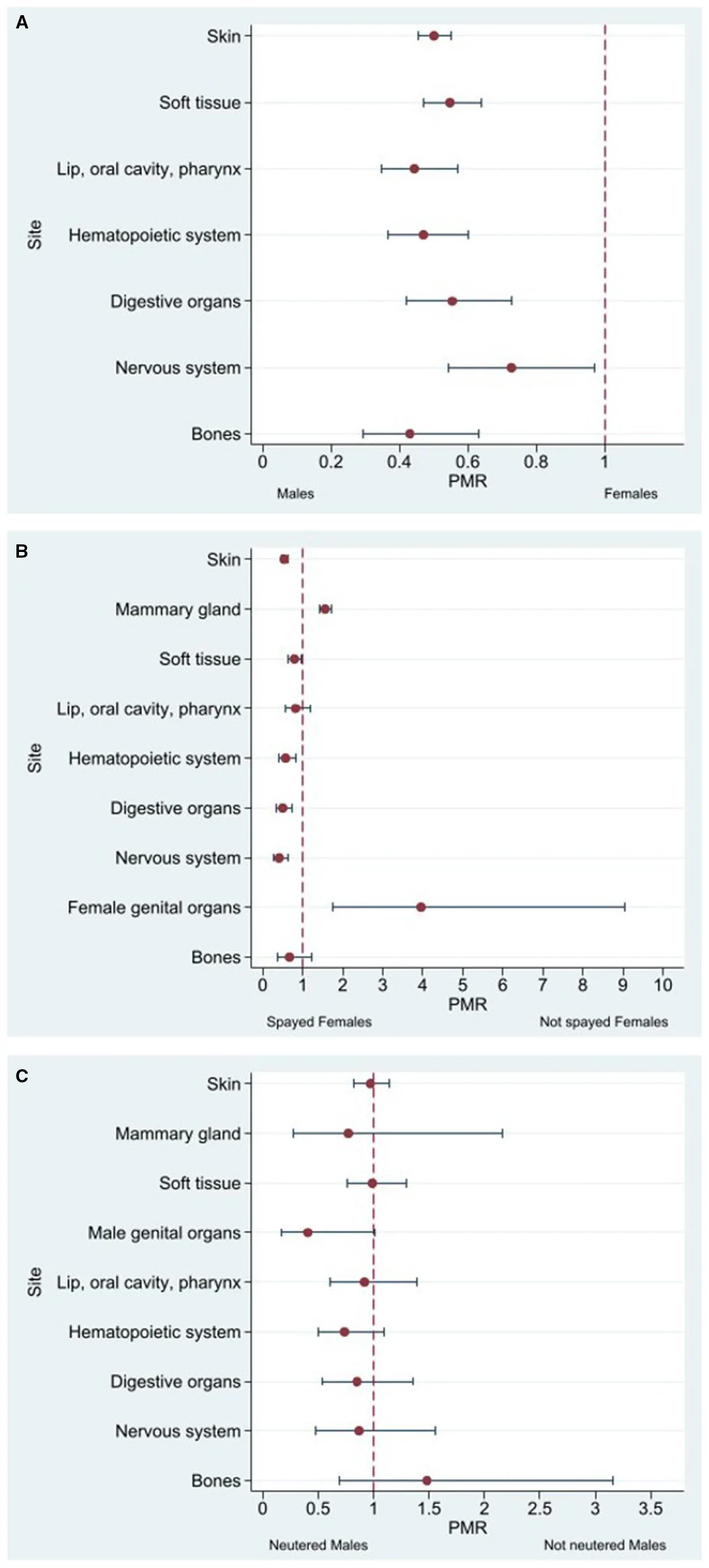
Age-adjusted proportional morbidity ratio (PMR) of malignant tumors for the top ten topographies for the categories: male and female dogs with male as reference category **(A)**; intact females and spayed females with neutered animals as reference category **(B)**; intact males and neutered males with neutered animals as reference category **(C)**.

The sex-stratified analysis (Figure 5A) shows that all the examined topographies are more frequent in males than in females, with the exception of mammary gland, for which a higher proportional frequency of diagnosis is observed in females compared to males (adj-PMR = 46.22; 95% CI: 29.75–71.81). Since the PMR value for mammary gland was markedly higher than that of all other sites, this topography was omitted from figure to preserve clear and balanced data representation. Moreover, intact females (Figure 5B) exhibit a higher number of female genital organs (adj-PMR = 3.96; 95% CI: 1.73–9.04) and mammary gland tumors compared to spayed females (adj-PMR = 1.56; 95% CI: 1.42–1.71). However, for hematopoietic (adj-PMR = 0.57; 95% CI: 0.39–0.83), skin (adj-PMR = 0.53; 95% CI: 0.46–0.62), digestive system (adj-PMR = 0.49; 95% CI: 0.33–0.74), and nervous system tumors (adj-PMR = 0.41; 95% CI: 0.28–0.62), spayed females appeared to have a higher proportional frequency. No significant differences were observed between intact and neutered males (Figure 5C). Graphical representation of the age-adjusted PMRs of categories related to sex and reproductive status are shown in Figure 5.

**Figure 6:**
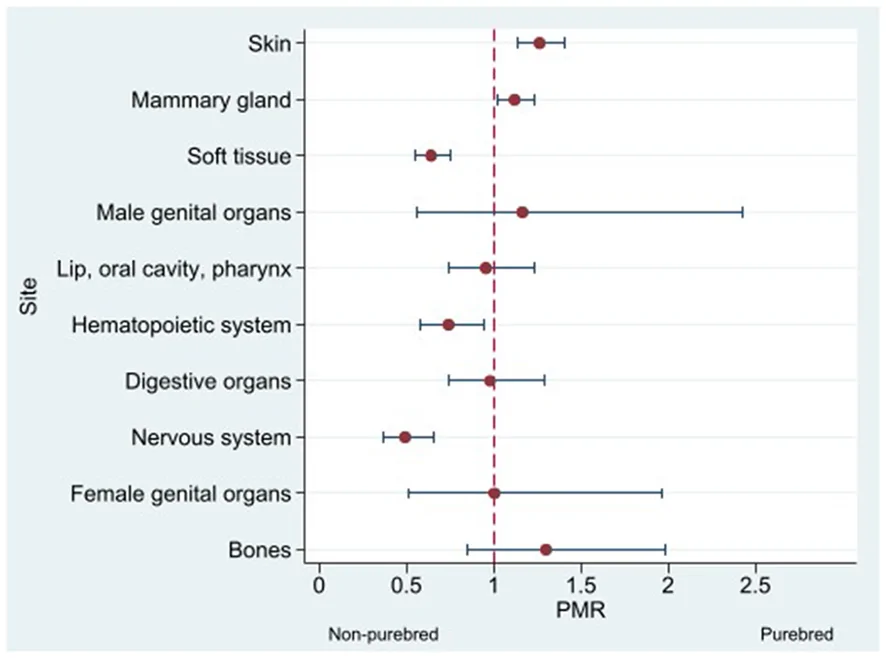
Age-adjusted proportional morbidity ratio (PMR) of malignant tumors for the top 10 topographies to compare purebred and not-purebred animals with not-purebred animals as reference category.

The comparison between purebred and not-purebred dogs (Figure 6) shows that purebred dogs exhibit a higher proportional frequency of skin (adj-PMR = 1.26; 95% CI: 1.13–1.40) and mammary gland tumors (adj-PMR = 1.12; 95% CI: 1.02–1.23). Conversely, purebred dogs show lower frequencies of diagnoses for hematopoietic system (adj-PMR = 0.74; 95% CI: 0.57–0.95), soft tissue (adj-PMR = 0.64; 95% CI: 0.54–0.75), and nervous system tumors (adj-PMR = 0.49; 95% CI: 0.36–0.65).

**Figure 7:**
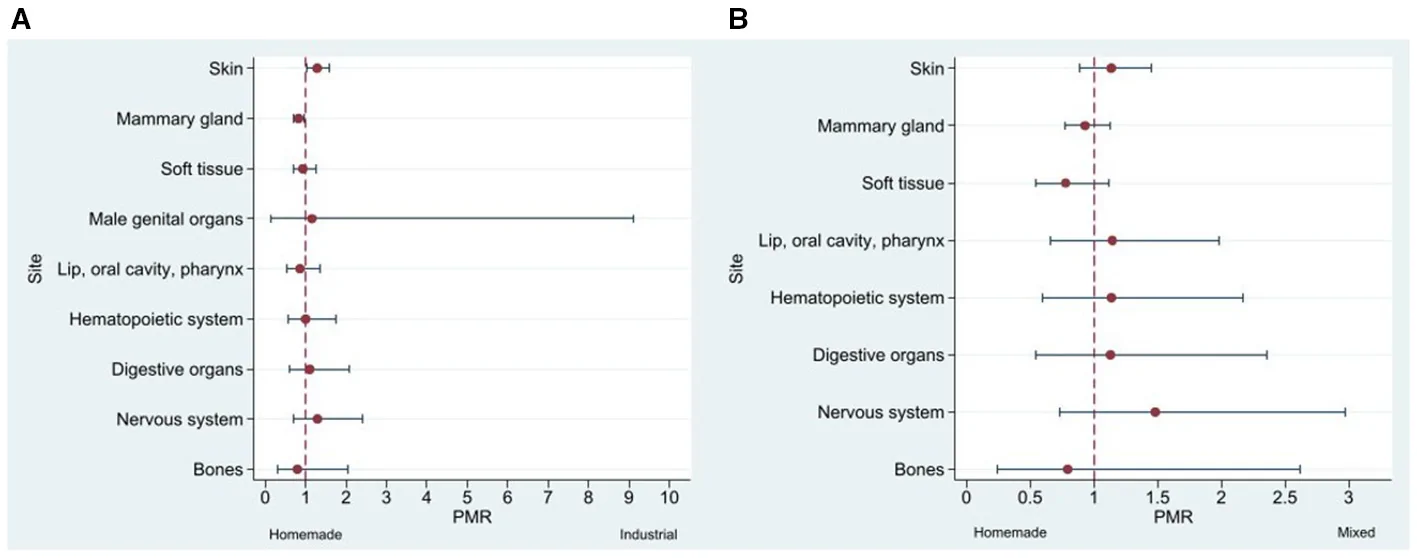
Age-adjusted proportional morbidity ratio (PMR) of malignant tumors for the top 10 topographies for the categories to compare diet habits: industrial vs. homemade **(A)** and mixed vs. homemade **(B)** with homemade diet as reference category.

Dogs fed industrial food show a higher proportional representation of skin tumors compared with those fed homemade diets (adj-PMR = 1.27; 95% CI: 1.03–1.57), whereas the frequency of mammary gland tumors appears slightly lower (adj-PMR = 0.81; 95% CI: 0.69–0.95) (Figure 7A). No significant differences were observed in the comparison between dogs fed mix of industrial and homemade food and dogs fed homemade diet (Figure 7B).

**Figure 8:**
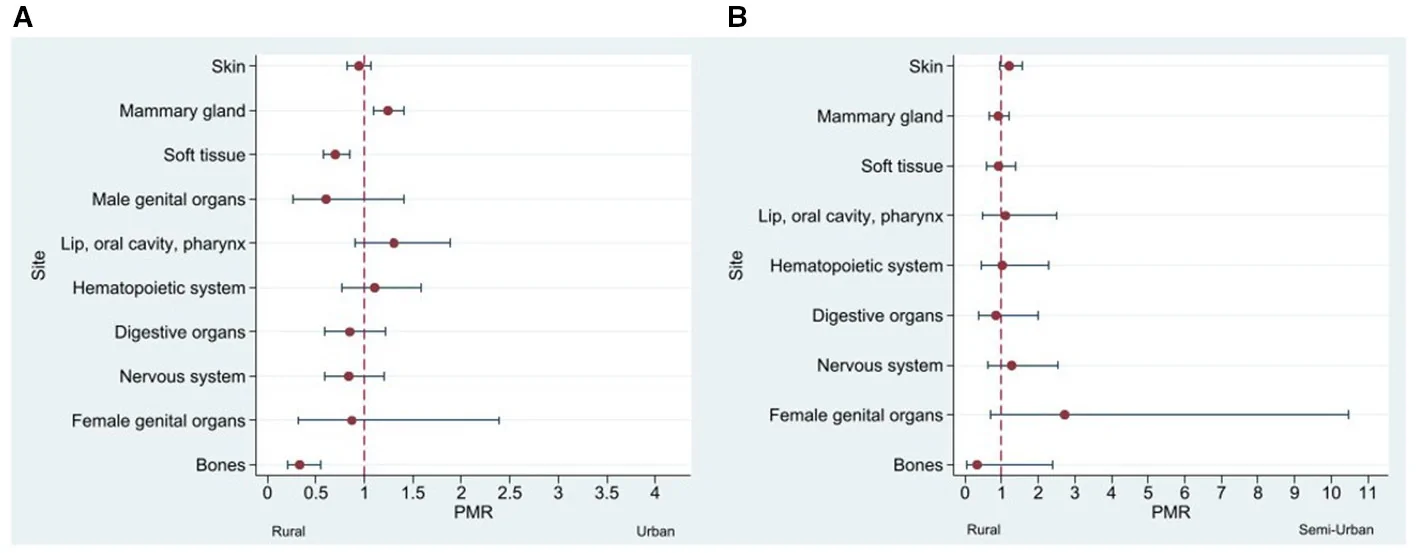
Age-adjusted proportional morbidity ratio of malignant tumors by topography to compare habitat: urban vs. rural **(A)** and semi-urban vs. rural **(B)** with rural as reference category.

**Figure 9:**
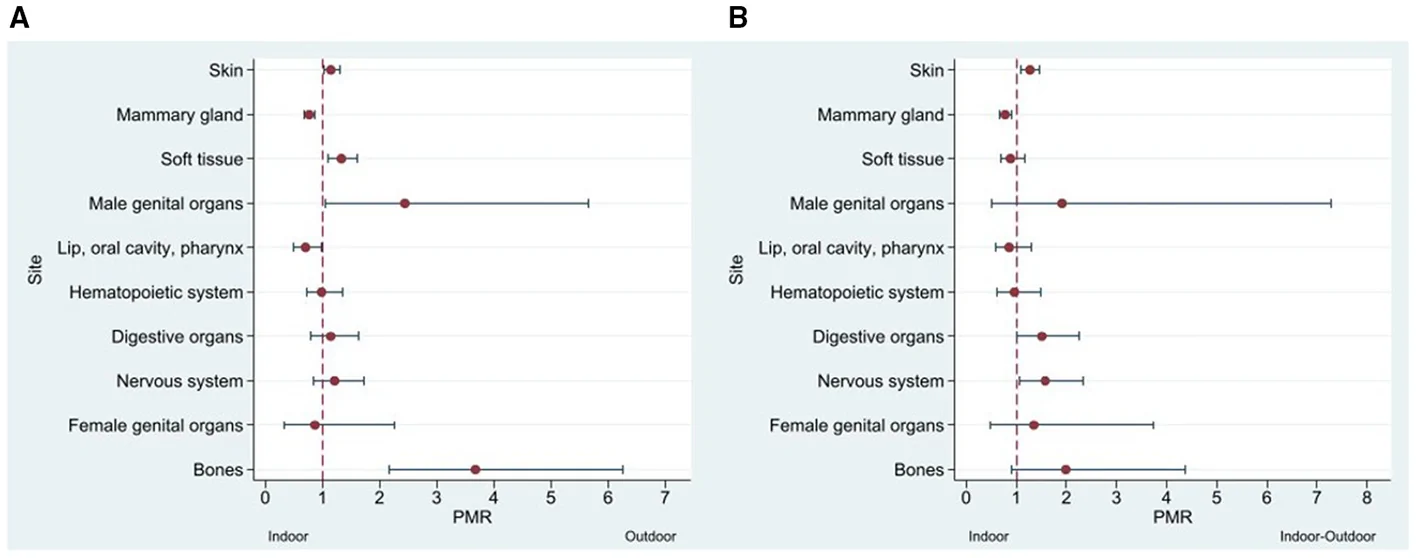
Age-adjusted proportional morbidity ratio of malignant tumors by topography to compare living environment: indoor vs. outdoor **(A)** and indoor/outdoor vs. indoor **(B)** with indoor as reference category.

Finally, regarding habitat (Figure 8) and living environment (Figure 9), dogs residing in urban areas show a higher proportional frequency of mammary gland tumors compared with those living in rural settings (adj-PMR = 1.24; 95% CI: 1.09–1.40), whereas the frequency of soft tissue tumors appears lower in urban areas (adj-PMR = 0.69; 95% CI: 0.57–0.84), as per bone tumors (adj-PMR = 0.33; 95% CI: 0.20–0.54). No significant differences were observed in the comparison between semi-urban habitat and rural habitat. Animals living exclusively outdoors show a higher proportional frequency of bone (adj-PMR = 3.67; 95% CI: 2.15–6.26), male genital organ (adj-PMR = 2.43; 95% CI: 1.05–5.64), soft tissue (adj-PMR = 1.33; 95% CI: 1.10–1.60), and skin tumors (adj-PMR = 1.14; 95% CI: 1.01–1.29) compared with those living solely indoors. Dogs with access to a garden similarly exhibit a higher proportional representation of skin (adj-PMR = 1.27; 95% CI: 1.09–1.47) and nervous system tumors (adj-PMR = 1.58; 95% CI: 1.06–2.34) compared with dogs kept exclusively indoors; latter results are borderline. Conversely, dogs living indoors seem to show a higher proportional representation of mammary gland tumor diagnosis compared with the other two groups.

**Figure 10:**
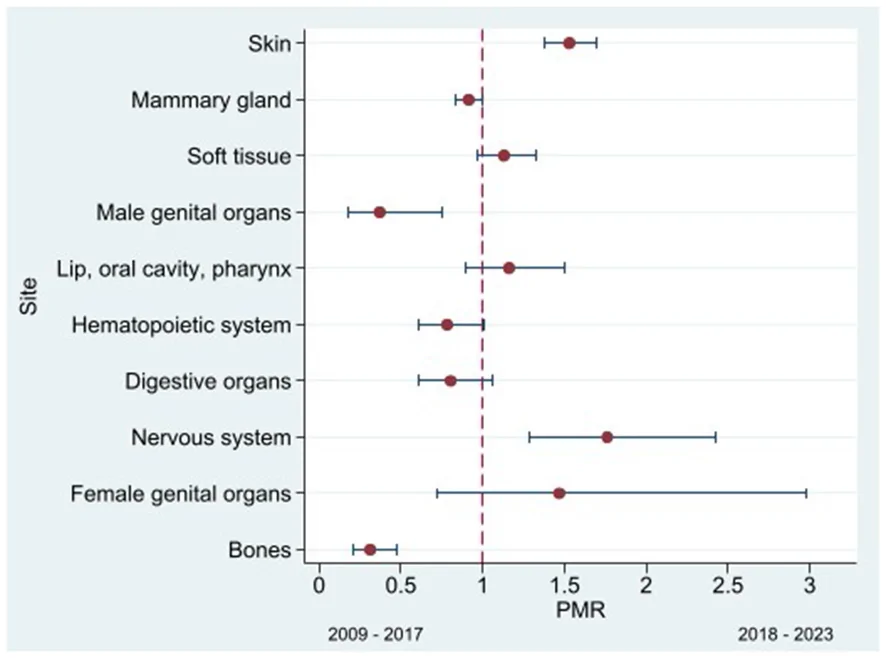
Age-adjusted proportional morbidity ratio of malignant tumors by topography to compare temporal period (2009–2017 vs. 2018–2023) with 2009–2017 as reference category.

When examining the temporal period (Figure 10), an increase in certain tumors is observed in the more recent years (2018–2023) compared with the earlier period, particularly for nervous system tumors (adj-PMR = 1.76; 95% CI: 1.28–2.42) and skin tumors (adj-PMR = 1.52; 95% CI: 1.37–1.70). Conversely, diagnoses of male genital organ tumors (adj-PMR = 0.36; 95% CI: 0.18–0.74) and bone tumors (adj-PMR = 0.31; 95% CI: 0.20–0.46) decreased in the more recent period. These patterns may reflect diagnostic or behavioral changes over time, although they do not allow for the establishment of a direct causal relationship.

### 3.3 Incidence estimate

In 2021, out of 744 active veterinary facilities in the Lazio Region, 430 were successfully contacted, and 52 structures participated in the survey, yielding a response rate of 12.1% among contacted practices. The total clinical volume across all structures was 9,043 canine visits per month. The crude (unweighted) average sample submission to IZSLT ratio was 21.8%, whereas the volume-weighted submission ratio, estimated based on annual survey visits, was 15.4% (16,662/108,516). Notably, the submission proportion to IZSLT for malignant tumors was over twice that of benign tumors, 24% (647/2,675) for malignant and 10% (443/4,426) for benign. The total number of tumors recorded by the ACR of Lazio in 2021 (1,090) was adjusted based on the weighted submission ratio (1,090/0.1535), resulting in an estimated total regional burden of 7,101 tumors in the canine population.

Applying the survey-derived proportions of tumors (62.3% benign and 37.7% malignant) to the estimated number, the total number of tumors resulted in 4,426 benign and 2,675 malignant tumors. These estimates were then used to calculate the corresponding incidence values, calculated on the adjusted total dog population of the Lazio Region (1,072,452), derived from the 807,556 dogs registered in the Regional Dog Database (28), which represents 75.3% of the total dog population (17). The resulting annual crude incidence estimates were:

- Malignant tumors: 249.4 per 100,000 dogs (95% CI: 240.08–259.05)
- Benign tumors: 412.7 per 100,000 dogs (95% CI: 400.65–425.01)
- Overall tumors: 662.1 per 100,000 dogs (95% CI: 646.87–677.66)

A sensitivity analysis was conducted to evaluate the effect of uncertainty related to survey-derived correction factors. Assuming alternative survey-derived sample submission rates ranging from 6.4% to 24.2%, the estimated overall crude incidence ranged from 419.5 to 1,570.9 cases per 100,000 dogs. Under the same assumptions, malignant tumor incidence ranged from 154.0 to 606.7 cases per 100,000 dogs, while benign tumor incidence ranged from 257.5 to 994.1 cases per 100,000 dogs. Detailed results are reported in Supplementary Tables S2–S4.

## 4 Discussion

The ACR of Lazio serves as a comprehensive regional depository, merging routine diagnostic data from the IZSLT with voluntary notifications from private practitioners across central Italy. Spatial analysis of the 15-year dataset revealed a highly skewed geographic pattern, with a small number of urban municipalities accounting for the vast majority of diagnoses. Rather than indicating localized biological risk, this distribution strongly mirrors regional human and canine demographics, alongside the density of veterinary infrastructure (9, 12). The metropolitan area of Rome, which hosts approximately 74.0% of the region's human population and the largest share of registered dogs, generated the highest volume of cases, followed by Latina, Viterbo, Frosinone, and Rieti. This gradient reflects a classic “catchment area” effect (13), where cases are naturally funneled toward superior diagnostic hubs. Therefore, these spatial patterns indicate where canine tumors are most readily detected and recorded rather than where intrinsic cancer risk is highest (24). Furthermore, the dramatic surge in case registrations observed after 2018 likely reflects the combined influence of several factors, including publicly funded diagnostic services, progressive consolidation of the cancer registry network, and increased engagement of veterinary professionals in case reporting, highlighting the importance of a multifaceted approach for building resilient, high-fidelity animal sentinel networks.

Anatomical distribution within the registry was heavily dominated by external, easily accessible topographies. Skin neoplasms were the most frequent topography (approximately 38.4%), a proportion that aligns well with the range reported in European literature including registries from Veneto (40.8%; (29)), Abruzzo and Molise (55.3%; (26)), and Switzerland (37.0%; (23, 30, 44)). Morphologically, adnexal tumors (29.1%) and mast cell tumors (26.6%) comprised over half of the cutaneous dataset, replicating diagnostic patterns reported in Japan, Portugal, Poland, and the Canary Islands (31–34). Swiss data reported by Graf et al. (35) likewise place mast cell tumors and follicular tumors among the predominant cutaneous morphologies, outlining a pattern highly comparable to that observed in our population.

Similarly, mammary gland tumors represented the second most common site, accounting for 23.2% of all diagnoses. This proportion is highly consistent with national multi-center data (24), Swiss cohorts (23) and Italian registries (9). Studies from the Canary Islands (34) describe a high burden of mammary tumors in female dogs and a predominance of malignant forms, a morphological pattern closely resembling that found in our dataset. Soft tissue tumors represent the third most frequent category (13.3%), dominated by lipomatous, vascular, and fibromatous neoplasms. This composition closely mirrors patterns observed in large German datasets (36), Swiss studies (35), and in recent Italian analyses (24, 26).

From a demographic perspective, the higher proportion of affected females (57.7%) is consistent with the substantial contribution of tumors arising in female reproductive tissues, especially mammary gland tumors, to the overall tumor burden observed in companion dogs, irrespective of geographical location (24, 37). The mean age of affected dogs in Lazio (8.8 years) closely matches values reported in Swiss registry and in studies from Japan, Germany, and the Canary Islands (31, 34, 36), where the peak frequency consistently falls between 8 and 11 years of age. This result reinforces the evidence that age is a well-established determinant of tumor occurrence in dogs, regardless of the geographical context.

The overwhelming predominance of superficial lesions (skin and mammary tissues exceeding 61.0% of the total dataset) underscores a prominent ascertainment and detection bias. Superficial and externally visible tumors are far more likely to be detected by owners and submitted by clinicians for diagnostic evaluation compared to internal neoplasms, which may remain undiagnosed or be detected only at advanced stages. Visceral and internal malignancies (e.g., hematopoietic, hepatic, or splenic tumors) are inherently under-reported because advanced imaging (CT/MRI) is required, and owners frequently opt for euthanasia without pursuing confirmatory histopathology or necropsy. This is further corroborated by the low necropsy rate (3.1%) within our registry.

Interestingly, malignant tumors accounted for 59.3% of cases in the ACR of Lazio, in line with studies from Italy, Croatia, and Japan, where malignant neoplasms were the most frequent ranging from 51% to 64.7% (26, 29, 38–40). Conversely, our 2021 survey-based estimation revealed that benign tumors actually dominate the real-world clinical landscape (62.3% benign vs. 37.7% malignant), as also reported by other authors (23). This discrepancy represents a notable phenomenon and reflects a clear submission bias: non-aggressive or overtly benign neoplasms are less frequently submitted for pathological examination by practitioners, leading to their artificial underrepresentation in laboratory-derived registry data.

To explore tumor patterns in the absence of population-based denominators for specific exposure variables, a proportional morbidity ratio (PMR) approach was adopted, as previously utilized in laboratory-derived veterinary surveillance (9, 26). As PMRs rely on the total number of cases collected rather than the general population at risk, they allow the exploration of associations and tumor distribution patterns across groups, while acknowledging inherent limitations in drawing definitive population-wide causal inferences. Therefore, PMRs should not be interpreted as measures of disease risk, but rather as indicators of differences in the distribution of tumor types among recorded cases. As with any registry-based analysis, residual confounding and reporting biases cannot be excluded.

Sex-stratified models demonstrated that male dogs carried a significantly higher proportion of malignant tumors across almost all non-sex-specific sites (e.g., oral cavity, skin, soft tissues, and hematopoietic system) compared to females. The comparison between neutered and intact males did not reveal significant differences in our analysis. In contrast, previous studies reported higher PMRs for malignant tumors of the male genital organs and lower PMRs across multiple anatomical sites in intact male dogs (9, 26). In particular, lower PMRs were observed for tumors affecting the eye, brain and meninges (9), as well as the blood and hematopoietic system, skin and subcutaneous tissues, oral cavity and pharynx, urinary organs, and bones, joints and cartilage (26). Conversely, intact females exhibited significantly higher PMRs for malignant mammary gland and reproductive organs tumors, in agreement with previous studies (9, 26). This pattern reflects the known hormone-dependence of these tumors, as observed in humans, with early ovariohysterectomy markedly reducing the risk (4), although recent evidence suggests that the association between spaying and reduced mammary tumor risk may be weaker or less consistent than previously assumed (41). Spayed females, however, showed higher proportional frequencies of malignant tumors affecting hematopoietic, skin, digestive, and nervous system, a pattern partially consistent with previous findings (9, 26). Unlike the results reported by Crescio et al. (9), we did not observe significant differences in PMRs between intact and spayed females for tumors of the lip, oral cavity, and pharynx, bones or for soft tissue.

Concerning breed, purebred dogs exhibit a statistically significant, higher age-adjusted PMRs for malignant tumors of skin topography and, similarly with Di Teodoro et al. (26), of mammary gland compared with not-purebred dogs. Conversely, purebred dogs showed lower PMRs for malignant tumors of hematopoietic, soft tissue, and nervous systems, in line with Crescio et al. (9). Discrepancies between these studies likely reflect differences in study design, population structure, case mixes, or the inherent limitations of registry-based data, particularly when using proportional measures that do not account for underlying population at risk. Differences in topographical classification across studies (e.g., grouping of skin, soft tissue and nervous system tumors in Di Teodoro et al. (26)) may partially limit direct comparability of results. Furthermore, the associations observed for reproductive status and breed should be interpreted cautiously, as residual confounding from factors not captured in the database (e.g., timing of neutering and other demographic or clinical characteristics) may persist despite age adjustment.

When evaluating diets and environmental exposures, the PMR analysis for diet showed a significantly higher age-adjusted PMR for malignant skin tumors in dogs fed industrial food compared to a homemade diet, whereas mammary gland malignant tumors showed lower age-adjusted PMR. These findings contrast with Di Teodoro et al. (26), who detected no significant dietary associations. Given the observational nature of the study, these associations should be interpreted cautiously and not as evidence of a causal effect of diet on tumor occurrence. Regarding habitat, the PMR for malignant mammary tumors was higher for dogs that lived in urban areas compared to those living in rural settings, whereas the PMRs for soft tissue and bones malignancies was lower in urban areas. Dogs living exclusively outdoors or having regular garden access showed higher PMRs for bone, male genital organs, soft tissue, and skin tumor sites compared to indoor dogs, potentially driven by chronic exposure to UV radiation and ambient environmental factors. Conversely, dogs living predominantly indoors showed a statistically significant, higher age-adjusted PMR for malignant mammary tumors, a finding likely explained by closer, more frequent physical monitoring by owners, leading to earlier detection. Finally, our data showed a significantly higher age-adjusted PMRs for malignant tumors affecting the skin (similarly to Crescio et al. (9)) and nervous system in the most recent years (2018–2023) compared with the earlier period, whereas the PMRs for malignant tumors of male genital organs and bones decreased. In contrast, Crescio et al. (9) reported increased PMRs for malignant tumors of male genital organs in the most recent period (2018–2021). These patterns may reflect diagnostic or behavioral changes over time rather than a direct shift in biological baseline risks.

While PMR analysis allowed relative comparisons across groups, incidence estimates were derived using survey-adjusted data to better capture the true burden of tumors in the reference population. The incidence estimates observed in this study fall within the wide range reported in the international literature, which spans several orders of magnitude depending on geographical area and study context, ranging from 282 to 1,948 cases per 100,000 dogs (8, 23, 29, 37, 39, 42). Our values are notably lower than those reported for insured dog populations in the United Kingdom, where incidence estimates can exceed 2,600 per 100,000 dogs due to seamless veterinary care access and mandatory diagnostic tracking tied to insurance payouts. On the other hand, our results are highly consistent with those reported in other Italian and European registry-based studies, where incidence estimates are generally lower, often ranging between approximately 200 and 900 cases per 100,000 dogs per year, reflecting differences in case detection, diagnostic intensity, and surveillance coverage. The method applied in this study is an additional approach to those previously used. As reported by Dhein et al. (23) and Fonti and Millanta (18), the variability of methodological approaches limits comparability between incidence estimates.

From a comparative “One Health” perspective, the human incidence of malignant tumors in Lazio Region (all topographies, males and females, all age classes) was estimated at 569.5 out of 100,000 individuals (ranging from 563.3 to 575.7) in 2021 (43). While direct cross-species mathematical comparisons are limited by biological differences and likely human underestimations due to temporary post-pandemic gaps in mortality surveillance (43), the general alignment of these data should be viewed as an opportunity for future comparative and integrated surveillance, highlighting the potential utility of the dog as a co-habitant sentinel.

A number of inherent limitations must be acknowledged in this registry-based study. First, because the ACR is a laboratory-based registry, the absolute caseload suffers from under-ascertainment due to the voluntary nature of sample submissions. This was highlighted by our survey, where 65.0% of the interviewed veterinary facilities reported not submitting specimens to the IZSLT laboratory. Second, the survey itself achieved a limited response rate (12.1%), which may restrict uniform territorial representativeness and introduce selection bias toward larger, oncology-focused practices. Third, responding facilities with higher workloads demonstrated a tendency to submit a lower proportion of samples, potentially driving an underestimation of the total tumor burden. Finally, a severe under-reporting bias was identified for benign lesions, which had a submission rate of just 10.0%, compared to 24.0% for malignant tumors.

Although these limitations are inherent to pathology-based registries, the integration of registry data with information obtained through the veterinary survey represents an attempt to quantify and partially correct for under-reporting, thereby improving the epidemiological usefulness of incidence estimates.

To assess the effect of uncertainty associated with survey-derived correction factors, a sensitivity analysis was performed. Although incidence estimates varied quantitatively across alternative assumptions, the main conclusions of the study were not materially affected.

To rigorously address these limitations, several advanced methodological strategies were applied. The adoption of a volume-weighted submission rate (15.4%) successfully corrected for the heterogeneous workloads of veterinary facilities, preventing smaller practices or highly active non-submitting practices from distorting the regional projection. Furthermore, separating the incidence estimations for malignant and benign tumors successfully mitigated the severe under-reporting of benign masses, neutralizing the submission bias of practitioners. Finally, integrating these survey-derived correction factors with a locally validated canine population completeness factor (75.3%) provided an unusually robust baseline denominator. While some residual regional variability cannot be entirely excluded, this model-based approximation represents a substantial methodological improvement over standard laboratory-derived veterinary datasets where population denominators are poorly defined or entirely missing.

Beyond the descriptive characterization of canine tumors in central Italy, this study proposes a practical framework for combining registry and survey data to improve incidence estimation in settings where complete case ascertainment is not achievable.

In veterinary medicine, the lack of standardized guidelines for data collection and reporting in animal cancer registries remains a recognized gap (18), highlighting the need for harmonized frameworks to improve data quality and interoperability. Nonetheless, growing evidence supports the relevance of comparative oncology as a multidisciplinary field contributing to both veterinary and human medicine (3, 5–7).

In this context, the ACR represents a pivotal tool for monitoring companion animal health, and integrated use of human and veterinary cancer registry data holds significant potential for identifying shared environmental risk factors and advancing public health initiatives within the One Health framework. In regions such as Lazio, where a human cancer registry is already established, future collaboration may enable the integration of animal and human data, facilitating comparative and epidemiological analyses in shared environments.

## 5 Conclusion

The ACR represents a valuable and necessary tool for collecting standardized data on spontaneous tumors in companion animals, supporting epidemiological research within the veterinary domain and enabling comparability across animal cancer registries at the international level. The quality and completeness of collected metadata are crucial to ensure accurate case enumeration and to strengthen studies investigating environmental risk factors from a One Health perspective within comparative oncology.

The present study contributes to the field of animal cancer epidemiology by providing additional evidence on tumor registration in dogs and proposing an approach for estimating tumor incidence that integrates registry and survey-based data.

## Data Availability

The data analyzed in this study is subject to the following licenses/restrictions: the datasets presented in this article are not readily available and are accessible upon reasonable request, subject to authorization. Requests to access these datasets should be directed to osservatorioepidemiologico@izslt.it.
