# Supplementary material for: The epidemiological landscape of canine tumors in central Italy: animal cancer registry insights, distribution patterns, and incidence estimates
Source: Front Vet Sci. 2026 Sep 8;13:1919722. doi: 10.3389/fvets.2026.1919722 (PMC13594995; doi:10.3389/fvets.2026.1919722)
Supplement: Supplementary file 1 [file Table_1.docx]

Supplementary Table S1. Distribution of the most frequent tumor diagnoses (≥1.00%) across anatomical topographic sites with an overall frequency ≥1.00% recorded in the canine ACR of Lazio, 2009-2023.

| **Tumor site** | **Tumor categories [code]** | **Tumors (N= 9387)** |
| --- | --- | --- |
| **Skin, n** |  | **3.767** |
|  | Adnexal and skin appendage neoplasms [839-842], n (%) | 1,096 (29.10%) |
|  | Mast cell neoplasms [974], n (%) | 1,001 (26.57%) |
|  | Basal cell neoplasms [809-811], n (%) | 462 (12.26%) |
|  | Neoplasms of histiocytes and accessory lymphoid cells [975], n (%) | 429 (11.39%) |
|  | Squamous cell neoplasms [805-808], n (%) | 395 (10.49%) |
|  | Melanocytoma and Melanomas [872-879], n (%) | 269 (7.14%) |
|  | Others, n (%) | 115 (3.05%) |
| **Mammary gland, n** |  | **2.275** |
|  | Complex mixed and stromal neoplasms [893-899], n (%) | 1,138 (50.02%) |
|  | Adenomas and adenocarcinomas [814-838], n (%) | 715 (31.43%) |
|  | Ductal and lobular neoplasms [850-854], n (%) | 238 (10.46%) |
|  | Epithelial neoplasms, NOS [801-804], n (%) | 67 (2.95%) |
|  | Squamous cell neoplasms [805-808], n (%) | 57 (2.51%) |
|  | Complex epithelial neoplasms [856-857], n (%) | 28 (1.23%) |
|  | Others, n (%) | 32 (1.40%) |
| **Soft tissues, n** |  | **1.310** |
|  | Lipomatous neoplasms [885-888], n (%) | 368 (28.09%) |
|  | Blood vessel tumors [912-916], n (%) | 360 (27.48%) |
|  | Fibromatous neoplasms [881-883], n (%) | 273 (20.84%) |
|  | Soft tissue tumors and sarcomas, NOS [880], n (%) | 152 (11.60%) |
|  | Neoplasms of histiocytes and accessory lymphoid cells [975], n (%) | 45 (3,44%) |
|  | Mast cell neoplasms [974], n (%) | 21 (1.60%) |
|  | Myxomatous neoplasms [884], n (%) | 17 (1.30%) |
|  | Osseous and chondromatous neoplasms [918-924], n (%) | 16 (1.21%) |
|  | Myomatous neoplasms [889-892], n (%) | 15 (1.15%) |
|  | Nerve sheath tumors [954-957], n (%) | 15 (1.15%) |
|  | Malignant lymphomas, NOS or diffuse [959-972], n (%) | 15 (1.15%) |
|  | Others, n (%) | 13 (0,99%) |
| **Male genital organs, n** |  | **575** |
|  | Specialized gonadal neoplasms [859-867], n (%) | 373 (64.87%) |
|  | Germ cell neoplasms [906-909], n (%) | 164 (28.52%) |
|  | Adenomas and adenocarcinomas [814-838], n (%) | 9 (1.57%) |
|  | Mast cell neoplasms [974], n (%) | 6 (1.04%) |
|  | Others, n (%) | 23 (4.00%) |
| **Lip, oral cavity, pharynx, n** |  | **499** |
|  | Odontogenic tumors [927-934], n (%) | 187 (37.48%) |
|  | Melanocytoma and Melanomas [872-879], n (%) | 112 (22.44%) |
|  | Squamous cell neoplasms [805-808], n (%) | 63 (12.63%) |
|  | Fibromatous neoplasms [881-883], n (%) | 41 (8.22%) |
|  | Soft tissue tumors and sarcomas, NOS [880], n (%) | 20 (4.01%) |
|  | Plasma cell neoplasms [973], n (%) | 16 (3.21%) |
|  | Blood vessel tumors [912-916], n (%) | 10 (2.00%) |
|  | Adenomas and adenocarcinomas [814-838], n (%) | 8 (1.60%) |
|  | Malignant lymphomas, NOS or diffuse [959-972], n (%) | 5 (1.00%) |
|  | Neoplasms of histiocytes and accessory lymphoid cells [975], n (%) | 5 (1.00%) |
|  | Others, n (%) | 32 (6.41%) |
| **Hematopoietic systems, n** |  | **273** |
|  | Blood vessel tumors [912-916], n (%) | 191 (69.96%) |
|  | Malignant lymphomas, NOS or diffuse [959-972], n (%) | 31 (11.35%) |
|  | Soft tissue tumors and sarcomas, NOS [880], n (%) | 14 (5.13%) |
|  | Lipomatous neoplasms [885-888], n (%) | 12 (4.40%) |
|  | Fibromatous neoplasms [881-883], n (%) | 9 (3.30%) |
|  | Neoplasms of histiocytes and accessory lymphoid cells [975], n (%) | 6 (2.20%) |
|  | Myomatous neoplasms [889-892], n (%) | 5 (1.83%) |
|  | Others, n (%) | 5 (1.83%) |
| **Digestive organs, n** |  | **245** |
|  | Adenomas and adenocarcinomas [814-838], n (%) | 99 (40.41%) |
|  | Malignant lymphomas, NOS or diffuse [959-972], n (%) | 25 (10.20%) |
|  | Adnexal and skin appendage neoplasms [839-842], n (%) | 19 (7.76%) |
|  | Cystic, mucinous and serous neoplasms [844-849], n (%) | 16 (6.53%) |
|  | Myomatous neoplasms [889-892], n (%) | 16 (6.53%) |
|  | Blood vessel tumors [912-916], n (%) | 16 (6.53%) |
|  | Soft tissue tumors and sarcomas, NOS [880], n (%) | 15 (6.12%) |
|  | Epithelial neoplasms, NOS [801-804], n (%) | 11 (4.49%) |
|  | Complex mixed and stromal neoplasms [893-899], n (%) | 10 (4.08%) |
|  | Plasma cell neoplasms [973], n (%) | 6 (2.45%) |
|  | Fibromatous neoplasms [881-883], n (%) | 5 (2.04%) |
|  | Others, n (%) | 7 (2.86%) |
| **Nervous system, n** |  | **197** |
|  | 954-957 Nerve sheath tumors | 192 (97.46%) |
|  | Others, n (%) | 5 (2.54%) |
| **Female genital organs,n** |  | **132** |
|  | Myomatous neoplasms [889-892], n (%) | 48 (36.36%) |
|  | Specialized gonadal neoplasms [859-867], n (%) | 27 (20.45%) |
|  | Adenomas and adenocarcinomas [814-838], n (%) | 20 (15.15%) |
|  | Fibromatous neoplasms [881-883], n (%) | 15 (11.36%) |
|  | Germ cell neoplasms [906-909], n (%) | 8 (6.06%) |
|  | Soft tissue tumors and sarcomas, NOS [880], n (%) | 3 (2.27%) |
|  | Epithelial neoplasms, NOS [801-804], n (%) | 2 (1.52%) |
|  | Squamous cell neoplasms [805-808], n (%) | 2 (1.52%) |
|  | Blood vessel tumors [912-916], n (%) | 2 (1.52%) |
|  | Others, n (%) | 5 (3.79%) |
| **Bones, n** |  | **114** |
|  | Osseous and chondromatous neoplasms [918-924], n (%) | 107 (93.86%) |
|  | Soft tissue tumors and sarcomas, NOS [880], n (%) | 3 (2.63%) |
|  | Others, n (%) | 4 (3.51%) |
