# Supplementary material for: The epidemiological landscape of canine tumors in central Italy: animal cancer registry insights, distribution patterns, and incidence estimates
Source: Front Vet Sci. 2026 Sep 8;13:1919722. doi: 10.3389/fvets.2026.1919722 (PMC13594995; doi:10.3389/fvets.2026.1919722)
Supplement: Supplementary file 2 [file Table_2.docx]

Supplementary Table S2. Sensitivity analysis of overall crude tumor incidence estimates under alternative assumptions for the survey-derived sample submission rate. Dog registry completeness was fixed at 75.3% in all scenarios.

| **Sample submission rate (%)** | **Overall crude incidence (per 100,000 dogs)** | **95% CI** |
| --- | --- | --- |
| 15.35 | 662.13 | [646.87 ; 677.66] |
| 6.47 | 1,570.89 | [1,547.44 ; 1,594.6] |
| 24.23 | 419.51 | [407.36 ; 431.92] |
