# Supplementary material for: The epidemiological landscape of canine tumors in central Italy: animal cancer registry insights, distribution patterns, and incidence estimates
Source: Front Vet Sci. 2026 Sep 8;13:1919722. doi: 10.3389/fvets.2026.1919722 (PMC13594995; doi:10.3389/fvets.2026.1919722)
Supplement: Supplementary file 3 [file Table_3.docx]

Supplementary Table S3. Sensitivity analysis of malignant tumor incidence estimates under alternative assumptions for survey-derived sample submission rates and malignant tumor proportions. Dog registry completeness was fixed at 75.3% in all scenarios.

| **Sample submission rate (%)** | **Malignant tumor proportion (%)** | **Malignant tumor incidence (per 100,000 dogs)** | **95% CI** |
| --- | --- | --- | --- |
| 15.35 | 37.67 | 249.43 | [240.08 ; 259.05] |
| 6.47 | 36.72 | 576.81 | [562.56 ; 591.32] |
| 6.47 | 38.62 | 606.65 | [592.04 ; 621.52] |
| 24.23 | 36.72 | 154.04 | [146.71 ; 161.64] |
| 24.23 | 38.62 | 161.97 | [154.44 ; 169.76] |
