# Supplementary material for: The epidemiological landscape of canine tumors in central Italy: animal cancer registry insights, distribution patterns, and incidence estimates
Source: Front Vet Sci. 2026 Sep 8;13:1919722. doi: 10.3389/fvets.2026.1919722 (PMC13594995; doi:10.3389/fvets.2026.1919722)
Supplement: Supplementary file 4 [file Table_4.docx]

Supplementary Table S4. Sensitivity analysis of benign tumor incidence estimates under alternative assumptions for survey-derived sample submission rates and benign tumor proportions. Dog registry completeness was fixed at 75.3% in all scenarios.

| **Sample submission rate (%)** | **Benign tumor proportion (%)** | **Benign tumor incidence (per 100,000 dogs)** | **95% CI** |
| --- | --- | --- | --- |
| 15.35 | 62.33 | 412.7 | [400.65 ; 425.01] |
| 6.47 | 61.38 | 964.24 | [945.83 ; 982.91] |
| 6.47 | 63.28 | 994.08 | [975.39 ; 1013.03] |
| 24.23 | 61.38 | 257.45 | [247.94 ; 267.22] |
| 24.23 | 63.28 | 265.47 | [255.82 ; 275.39] |
